# Suppressors of selection

**DOI:** 10.1371/journal.pone.0180549

**Published:** 2017-07-10

**Authors:** Fernando Alcalde Cuesta, Pablo González Sequeiros, Álvaro Lozano Rojo

**Affiliations:** 1 Departamento de Matemáticas, Universidade de Santiago de Compostela, E-15782 Santiago de Compostela, Spain; 2 Departamento de Didácticas Aplicadas, Facultade de Formación do Profesorado, Universidade de Santiago de Compostela, Avda. Ramón Ferreiro 10, E-27002 Lugo, Spain; 3 Centro Universitario de la Defensa, Academia General Militar, Ctra. Huesca s/n. E-50090 Zaragoza, Spain; 4 IUMA, Universidad de Zaragoza, Pedro Cerbuna 12, E-50009 Zaragoza, Spain; 5 GeoDynApp - ECSING Group, Spain; Universitat Rovira i Virgili, SPAIN

## Abstract

Inspired by recent works on evolutionary graph theory, an area of growing interest in mathematical and computational biology, we present examples of undirected structures acting as suppressors of selection for any fitness value *r* > 1. This means that the average fixation probability of an advantageous mutant or invader individual placed at some node is strictly less than that of this individual placed in a well-mixed population. This leads the way to study more robust structures less prone to invasion, contrary to what happens with the amplifiers of selection where the fixation probability is increased on average for advantageous invader individuals. A few families of amplifiers are known, although some effort was required to prove it. Here, we use computer aided techniques to find an exact analytical expression of the fixation probability for some graphs of small order (equal to 6, 8 and 10) proving that selection is effectively reduced for *r* > 1. Some numerical experiments using Monte Carlo methods are also performed for larger graphs and some variants.

## Introduction

Evolutionary dynamics has been classically studied for well-mixed populations, but there is a wide interest in the evolution of complex networks after site invasion. The process transforming nodes occupied by residents into nodes occupied by mutants or invaders is described by the *Moran model*. Introduced by Moran [1] as the Markov chain counting the number of invading mutants in a well-mixed population, it was adapted to weighted graphs by Lieberman et al. [2] and Nowak [3] (see also [4–8]). For undirected networks where links have no orientation, invaders will either become extinct or take over the whole population, reaching one of the two absorbing states, *extinction* or *fixation*. The *fixation probability* is the fundamental quantity in the stochastic evolutionary analysis of a finite population.

If the population is well-mixed, at the beginning, one single node is chosen to be occupied by an invader individual among a population of *N* resident individuals. Afterwards, an individual is randomly chosen for reproduction, with probability proportional to its reproductive advantage (1 for residents and *r* ≥ 1 for invaders), and its clonal offspring replaces another individual chosen at random. In this case, the fixation probability is given by

$$\begin{array}{c} \Phi_{0}\left(r\right)=\frac{1-r^{-1}}{1-r^{-N}}=\frac{r^{N-1}}{r^{N-1}+r^{N-2}+\cdots +r+1}. \end{array}$$ (1)

In evolutionary network theory, the nodes are occupied by resident or invader individuals (usually assuming that the invader arises uniformly at random) and the replacements are limited to the nodes which are connected by oriented links. As in the well-mixed case, we also restrict ourselves to birth-death updating when the process evolves. According to the Circulation Theorem [2], any weight-balanced network has the same fixation probability as the well-mixed population of the same size *N*. In the undirected case, this means that the *temperature* *T*_*i*_ = ∑_*j*∼*i*_ 1/*d*_*j*_ of every vertex *i* (where *j* is a neighbor of *i* and *d*_*j*_ is the number of neighbors of *j*) is constant, and the network is said to be *isothermal*. But there are graph structures altering substantially the behavior of the fixation probability depending on the fitness. For example, the (average) fixation probability in the oriented line is equal to 1/*N* and the reproductive advantage of the invader individuals is completely suppressed. But in the directed case, absorbing barriers may not be accessible from any state, and the fixation probability may be even null (see [9] for an example).

**Fig 1:**
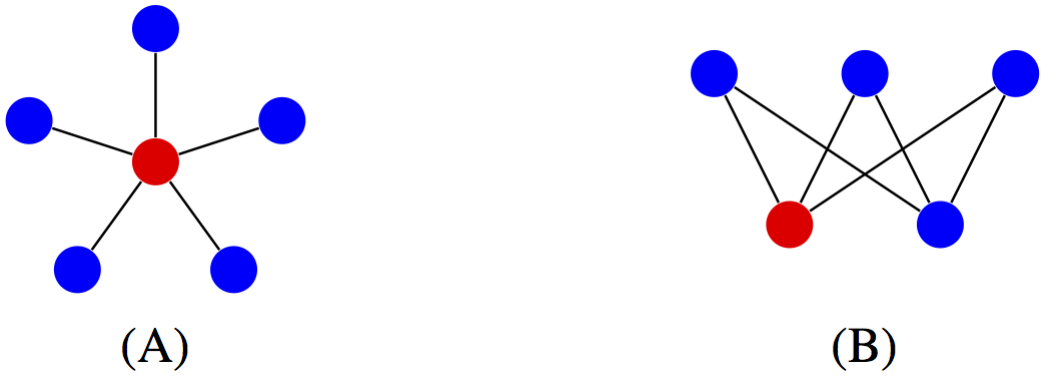
Star and complete bipartite graphs. (A) In the star graph *K*_1,*m*_, the center is connected with *m* peripheral nodes. (B) The vertex set of a complete bipartite graph *K*_*n*,*m*_ is divided into two disjoint sets interconnected by edges.

Thus, we focus our attention on connected undirected networks where absorbing barriers can be reached from any state. As showed in [2, 3] (see also [10]), there are directed and undirected graph structures that asymptotically amplify this advantage. The fixation probability of a complete bipartite network *K*_*N*−*m*,*m*_ (described in Fig 1) converges to the same limit as the fixation probability

$$\begin{array}{c} \Phi_{2}\left(r\right)=\Phi_{0}\left(r^{2}\right)=\frac{1-r^{-2}}{1-r^{-2N}} \end{array}$$ (2)

of the Moran process with fitness *r*^2^ as *m* → ∞ and *N* − *m* is constant [9]. Assuming that fitness differences are amplified or reduced for network sequences of increasing size, a notion of *amplifier* and *suppressor of selection* has been introduced in [10] for several initialization types (describing the initial distribution of the invasion process). To distinguish both dynamics, that is, amplification and suppression of selection, a numerical analysis for a few fitness values *r* = 0.75, 1, 1.25, 1.5, 1.75 has been done in [11] for birth-death and death-birth processes on directed and undirected graphs (see [12] for a comparative analysis of both update mechanisms).

Here we always assume that the distribution is uniform: the probability that a node will be occupied by the initial invader is equal for all the nodes, see Eq 7. We say that a network is an *amplifier of selection* if the fixation probability Φ(*r*) > Φ_0_(*r*) and a *suppressor of selection* if Φ(*r*) < Φ_0_(*r*) for all *r* > 1. Notice that Φ(1) = 1/*N* and the inequalities must be reversed for *r* < 1. Due to the exact analytical expression given by Monk et al. [13] using martingales (see also [10]), one can see that star graphs and complete bipartite graphs are amplifiers of natural selection whose fixation probabilities are bounded from above by Φ_2_(*r*). The existence of suppressors of selection was firstly showed in [14], but similarly to [11] only for some fitness values (namely, for values *r* ≤ 10). The aim of the paper is to present the first examples of graphs (of order 6, 8 and 10) which are suppressors of selection for any fitness value *r* > 1. From the point of view of robustness against invasion [15], these graphs are more robust than complete graphs (being now necessary to add a sign to ‖Φ − Φ_0_‖_∞_ = sup_*r*≥1_|Φ(*r*) − Φ_0_(*r*)|). Better yet, we propose a complete family of graphs of even order 2*n* + 2 with *n* ≥ 2, called *ℓ-graphs*, which we believe are suppressors of selection. The proof of this assertion for the graphs of order 6, 8 and 10 is completed with a numerical simulation for larger orders. Some other variants are also explored numerically in order to understand why they are suppressors of selection.

## Results

All the examples of so-called suppressors of selection given in [2, 3] are directed graphs. The abundance of amplifiers and suppressors of selection have been explored numerically by Hindersin et al. in [11] for directed and undirected graphs under birth-death and death-birth updating. Different types of initialization or placement of new invaders have been distinguished in [10] in order to classify different evolutionary dynamics on directed graphs. As explained, we focus our attention on connected undirected graphs under uniform initialization.

**Fig 2:**
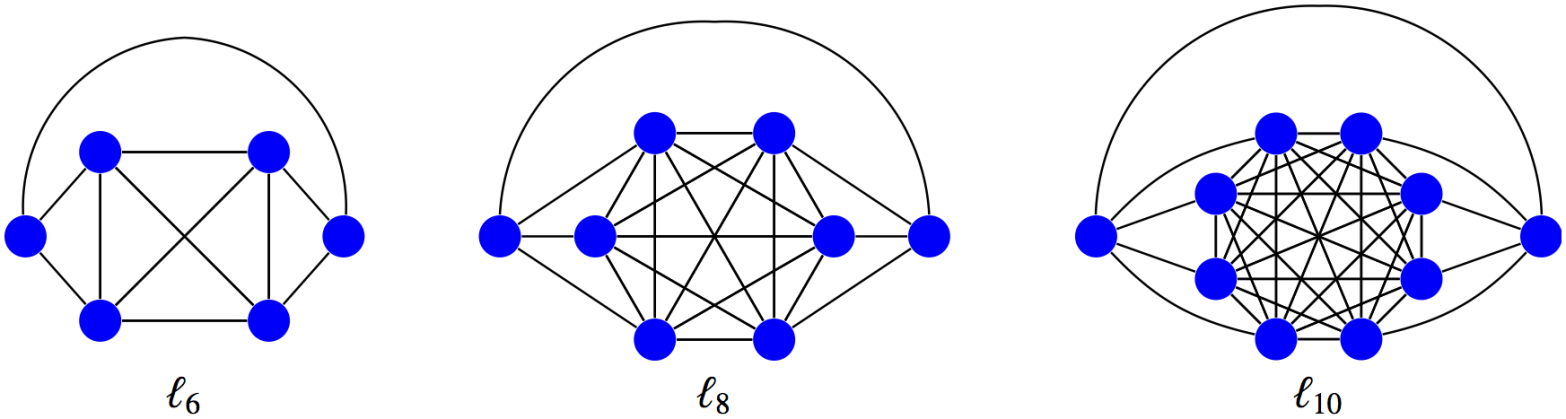
Graphs of order 6, 8 and 10 in the *ℓ*-family.

Firstly, we computed the fixation probability of all undirected graphs of order 10 or less for fitness values *r* varying from 0.25 to 10 with step size of 0.25 using with the *FinisTerrae2* supercomputer (1024 cores of Haskell 2680v3 CPUs for almost 3 days) installed at CESGA, [16]. We found an unique suppressor of selection of order 6, namely the graph *ℓ*_6_, although there are other possible suppressors in orders varying from 7 to 10. We constructed the graphs *ℓ*_8_ and *ℓ*_10_ (as well the whole *ℓ*-family) from this initial example. More precisely, we call *ℓ-graph* an undirected graph of even order *N* = 2*n* + 2 ≥ 6 obtained from the complete graph *K*_2*n*_ by dividing its vertex set into two halves with *n* ≥ 2 vertices and adding 2 extra vertices. Each of them is connected to one of the halves of *K*_2*n*_ and with the other extra vertex. Graphs *ℓ*_6_, *ℓ*_8_ and *ℓ*_10_ of order 6, 8 and 10 are shown in Fig 2.

### Graphs *ℓ*_6_, *ℓ*_8_ and *ℓ*_10_ are suppressors of selection

Computer aided techniques has been used to find exact analytical expressions of the fixation probability Φ for the first elements of this family with orders 6, 8 and 10 (see Fig 2). This computation proves that the graphs *ℓ*_6_, *ℓ*_8_ and *ℓ*_10_ are suppressors of selection with fixation probabilities Φ(*r*) < Φ_0_(*r*) for all *r* > 1 and Φ(*r*) > Φ_0_(*r*) for all *r* < 1.

At first, to bound the fixation probability from above, one could try to stop the process on *ℓ*_2*n*+2_ at the time that some extra vertex is occupied by a mutant. But as we will see later, the evolution from that time on seems to play an essential role in determining the suppressor character of the graph. Like for star and looping star graphs, which are amplifiers of selection for uniform initialization [10], we needed then to find the exact analytical expression of the fixation probability. Unfortunately, the elegant martingale method proposed in [13, Theorem 2.1] and later used in [10] (which is based on Doob’s optional stopping theorem [17]) is not useful for *ℓ*_6_, *ℓ*_8_ and *ℓ*_10_. We have had to implement a specific method to compute exactly their fixation probability.

As we shall see in the description of the mathematical model in the Methods section, the fixation probability Φ(*r*) is a rational function given as the quotient of two rational polynomials Φ′(*r*) and Φ′′(*r*) of degree bounded above by 2^*N*^ − 2. Using the symmetries of each *ℓ*-graph, we can lower this bound to a quantity

$$\begin{array}{c} d=\frac{N(N+1)}{2}-2\ll 2^{N}-2, \end{array}$$ (3)

(as proved in the Methods section and in S1 Text) and hence there are at most 2(*d* + 1) coefficients involved in Φ(*r*). Since Φ(*r*) converges to 1 as *r* → +∞, the leading coefficients of Φ′(*r*) and Φ′′(*r*) can be assumed to be 1 and that number is reduced to 2*d*. Then we can replace the system of 2^*N*^ linear equations defining the fixation probability Φ(*r*) (see Eq 6) with a system of 2*d* linear equations (see Eq 8) corresponding to the 2*d* rational coefficients of Φ′(*r*) and Φ′′(*r*), which arise from evaluating Φ(*r*) for integer and rational values of the fitness *r* varying from 1 to *d* + 1 and from 1/2 to 1/*d*. Finally, we wrote a SageMath program [18] (see S1 File) to symbolically compute the exact fixation probability Φ(*r*) of the graphs *ℓ*_6_, *ℓ*_8_ and *ℓ*_10_ for these fitness values and then to solve the reduced linear system. Once the fixation probability Φ has been calculated, the sign of the difference Δ = Φ − Φ_0_ is analyzed to confirm that Δ(*r*) < 0 for all *r* > 1. In the Methods section, we give a more detailed explanation of both theoretical and computational arguments used to have exact analytical expressions of the fixation probability Φ for *ℓ*_6_, *ℓ*_8_ and *ℓ*_10_. The exact values of Φ and Δ are given in S1 Text. As a result of the uniform initialization, since 2/*N* converges to 0 as *N* = 2*n* + 2 goes to infinity, the fixation probabilities Φ(*r*) of *ℓ*_*N*_ and Φ_0_(*r*) of *K*_*N*_ given by Eq 1 become more and more closer and the suppression effect tends to disappear for large populations. Notice however that this phenomenon can be avoided by modifying the initialization type or increasing the number of extra vertices.

### Numerical experiments in larger orders. Further examples

**Fig 3:**
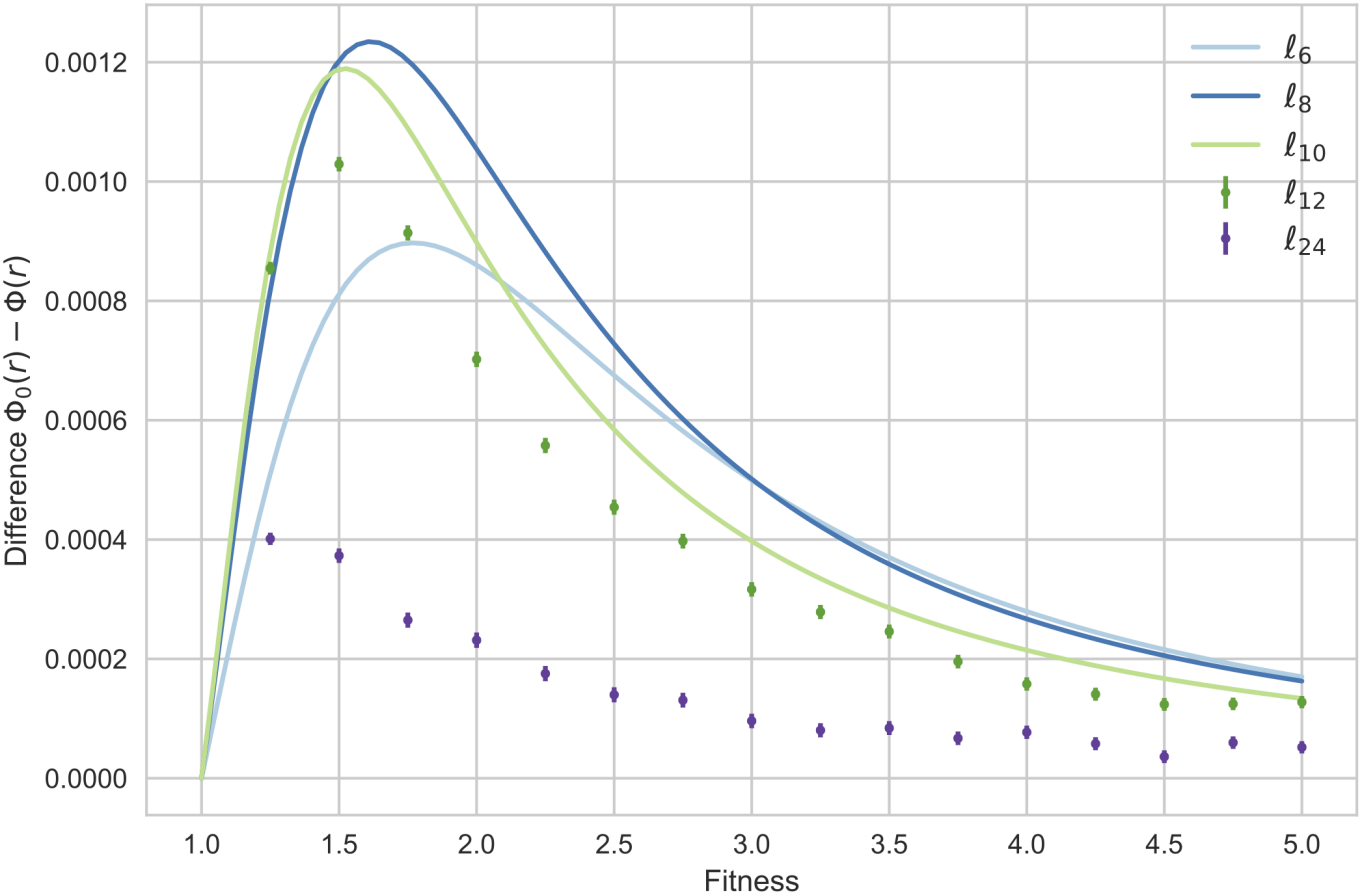
The exact differences Φ_0_(*r*) − Φ(*r*) for *ℓ*_6_, *ℓ*_8_ and *ℓ*_10_ and some estimates for *ℓ*_12_ and *ℓ*_24_. The functions Φ_0_(*r*) − Φ(*r*) associated to the *ℓ*-graphs of order 6, 8, and 10 are represented for fitness values *r* varying from 1 to 4. For the *ℓ*-graphs of order 12 and 24, we applied the Monte Carlo method to compute the difference between the fixation probabilities of each graph and the complete graph of the same order using 10^10^ trials for each fitness value *r* varying from 0 to 4 with step size of 0.25. A 99% confidence interval is showed for each simulated value.

However, the method used for *ℓ*_6_, *ℓ*_8_ and *ℓ*_10_ does not seem applicable to larger orders since it would require a substantial amount of memory and computation time. Therefore, we explored the suppression of selection for other graphs in the *ℓ*-family using Monte Carlo simulation (applying the *Loop-Erasing* technique of [9] to speedup the computations). Even if it does not require much memory and can be parallelized on a computer cluster, a very large number of trials—namely 10^10^ trails for each fitness value—has been necessary to compare the fixation probability of *ℓ*_12_ and *ℓ*_24_ with that of the complete graphs of the same order. In fact, since the fixation probabilities Φ(*r*) and Φ_0_(*r*) become more and more closer, we should need to increase this number more and more as *N* goes to ∞. Anyway, for fitness values *r* varying from 0 to 4 with step size of 0.25, we showed that the *ℓ*-graphs of orders 12 and 24 are also suppressors of selection as can be seen in Fig 3. In S1 Fig, a similar method is applied to all the graphs *ℓ*_2*n*+2_ with 2 ≤ *n* ≤ 11, including *ℓ*_6_, *ℓ*_8_ and *ℓ*_10_ in order to compare the fixation probabilities obtained by symbolic computation with the numerical solutions given by Monte Carlo simulation.

**Fig 4:**
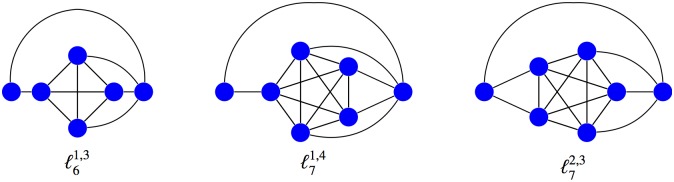
Unbalanced *ℓ*-graphs of order 6 and 7. The exponents are the sizes of the partition of the central *K*_*n*_.

**Fig 5:**
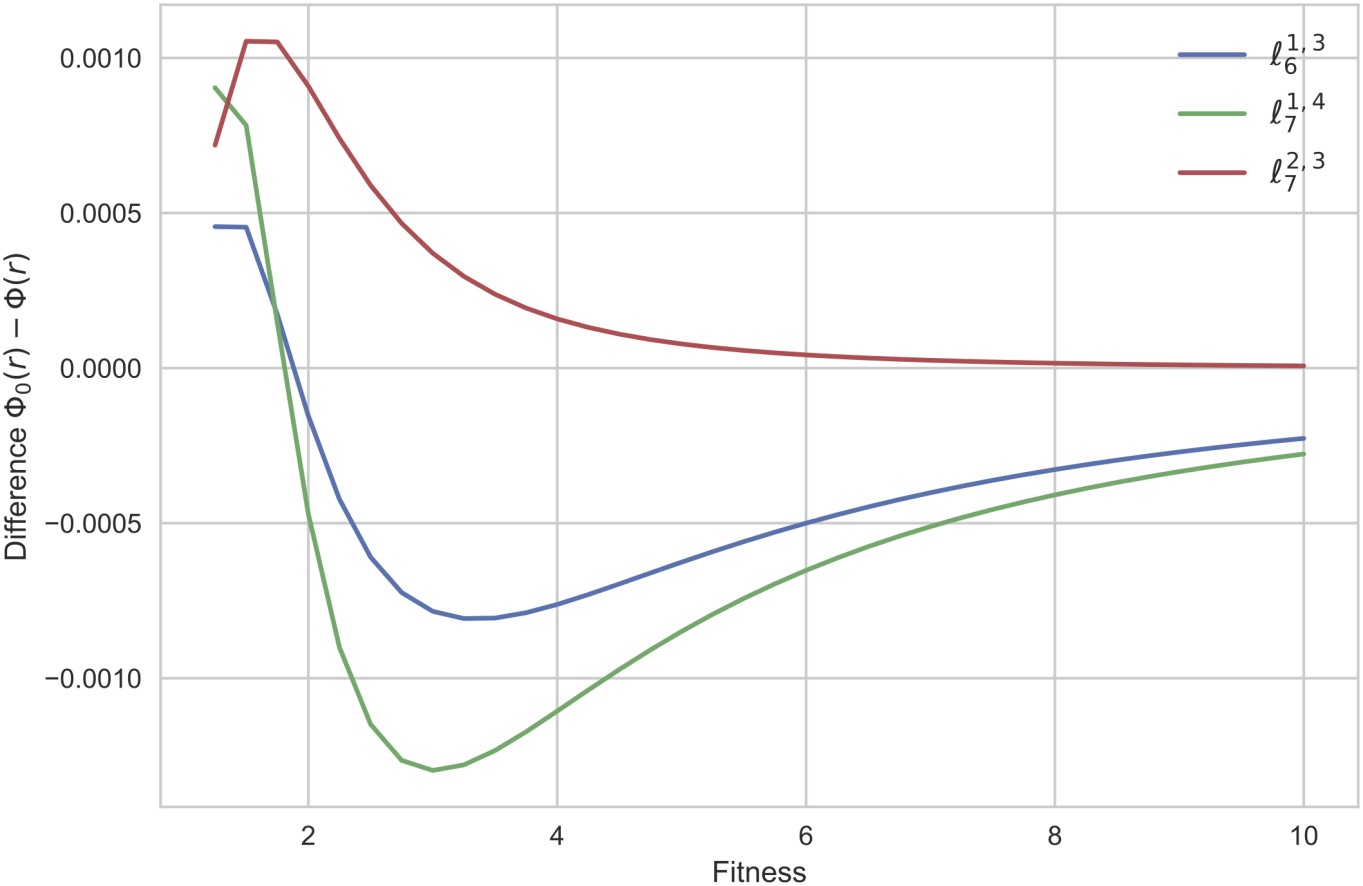
The differences Δ(*r*) = Φ(*r*) − Φ_0_(*r*) for the unbalanced graphs $\ell_{6}^{1,3}$, $\ell_{7}^{1,4}$, and $\ell_{7}^{2,3}$. The differences Δ(*r*) = Φ(*r*) − Φ_0_(*r*) have been estimated using 10^10^ trials for each fitness value *r* varying from 0 to 10 with step size of 0.25.

**Fig 6:**
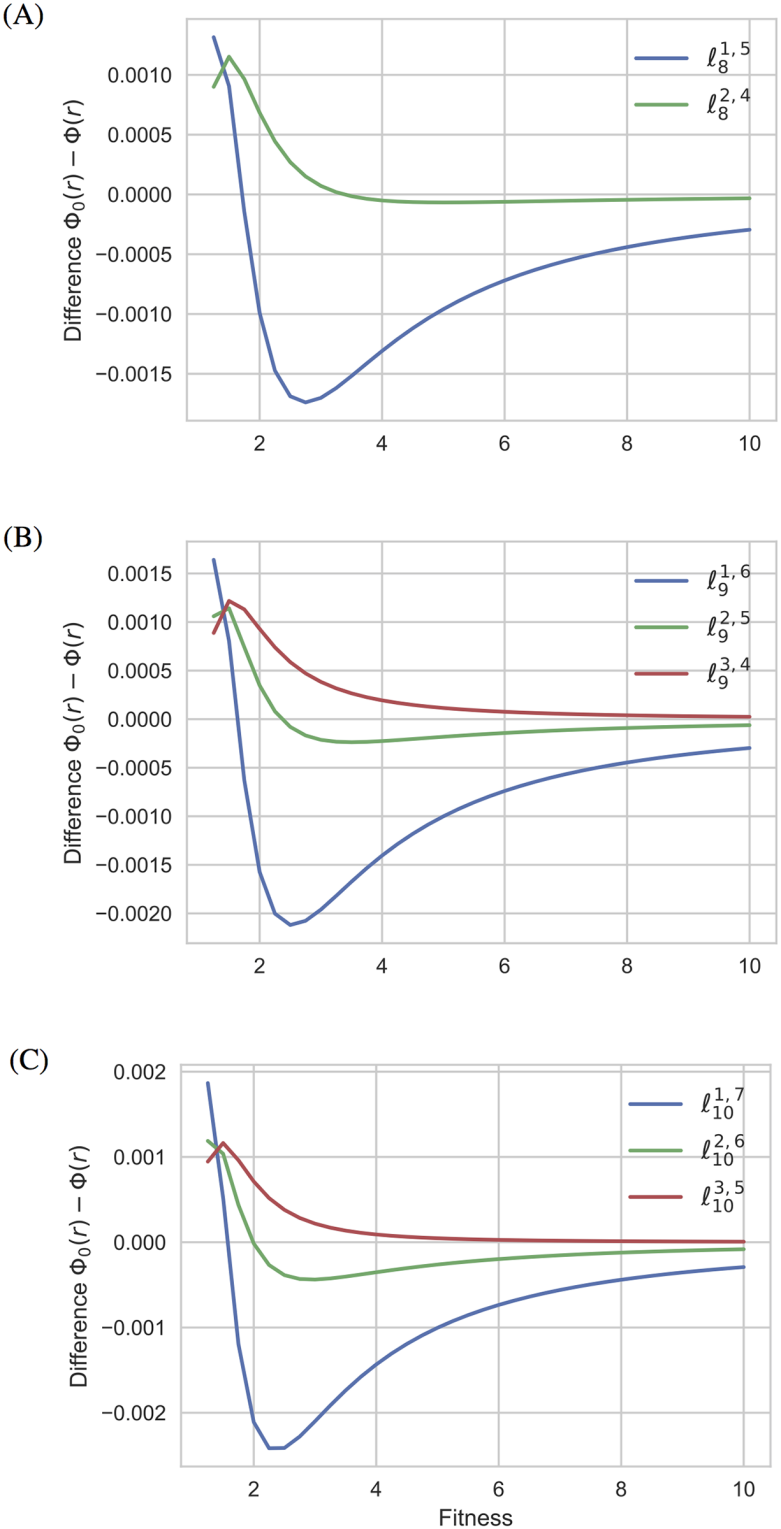
The differences Δ(*r*) = Φ(*r*) − Φ_0_(*r*) for the unbalanced graphs of order 8, 9, and 10. Unbalanced *ℓ*-graphs of (A) order 8, (B) order 9 and (C) order 10.

To investigate the structural reasons of the suppression of selection in these graphs, this experiment has been completed by altering the balance in the connections of the two extra nodes with the central complete graph in order 6 and considering two variants (*a fortiori* unbalanced) of order 7 (see Fig 4). As showed in Fig 5, the graphs $\ell_{6}^{1,3}$ and $\ell_{7}^{1,4}$ become amplifiers of selection from relatively small values of the fitness, while the graph $\ell_{7}^{2,3}$ is a suppressor of selection for high fitness values. We discover a similar behavior for larger orders (see Fig 6).

## Discussion

Motivated by interest in the robustness of networks against invasion, we tried to shed some light on the influence of the structural properties of graphs upon increasing or decreasing the fixation probability of new invaders occupying the nodes of a network. We computed the fixation probability of all undirected graphs of order 10 or less for fitness values *r* varying from 0.25 to 10 with step size of 0.25 using the *FinisTerrae2* supercomputer (1024 cores of Haskell 2680v3 CPUs for almost 3 days) installed at CESGA [16]. Thanks to this experimental approach, we found that there are graph structures acting as suppressors of selection according to the terminology introduced in [2, 3]. This means that, for every fitness value *r* > 1, the average fixation probability Φ(*r*) of an advantageous invader individual placed at a random node is strictly less than that of this individual placed in a well-mixed population. For neutral drift *r* = 1, both probabilities Φ(1) and Φ_0_(1) are obviously equal, whereas the average fixation probability Φ(*r*) becomes strictly greater than Φ_0_(*r*) for a disadvantageous invader with fitness *r* < 1. We proposed a novel method to compute the fixation probability of graphs having low order and a big group of symmetries, and we used computer aided techniques to find an exact analytical expression of the fixation probability for three examples of size 6, 8 and 10. The SageMath program [18] used to compute the fixation probability of these graphs is available at [19]. Monte Carlo simulation was also used to see with high precision that other graphs in this family are suppressors of selection for some fitness values (varying from 1 to 4 with step size of 0.25). Memory requirements make it unfeasible to apply the same method for large orders, but it could be useful to study transitions between both regimes, suppression and amplification, in low order. On the other hand, although we are only concerned here with the evolutionary dynamics of graphs under birth-death updating, similarly to the work by Kaveh et al. [12] and Hindersin et al. [11], it could be also interesting to study the properties of the *ℓ*-family under death-birth updating. We also showed that the mechanism that activates the suppression of selection is quite subtle, since a certain imbalance in the number of nodes of the central complete graph which are connected with each additional node transforms our models into amplifiers from certain fitness values. Finally, if the spreading of favorable innovations can be enhanced by those network structures amplifying the advantage of mutant or invader individuals [20], as counterpart, the discovery of these examples is a first step towards finding structural properties that increase the robustness of a complex network against invasion [15]. This is a particularly interesting property for biological networks like brain and protein-protein interaction networks, as well as for technological networks like electrical power grids or backbone networks, where high fitness values are possible. In fact, these kind of models have had impact not only in evolutionary and invasion dynamics, but also in tumor growth [3, 21, 22] and economics and management [23].

## Methods

### Mathematical model

Let *G* be a connected undirected graph with node set *V* = {1, …, *N*}. Denote by *d*_*i*_ the degree of the node *i*. The *Moran process* on *G* is a Markov chain *X*_*n*_ whose states are the sets of nodes *S* inhabited by mutant or invader individuals at each time step *n*. The transition probabilities are obtained from a stochastic matrix *W* = (*w*_*ij*_) where *w*_*ij*_ = 1/*d*_*i*_ if *i* ∼ *j* are neighbors and *w*_*ij*_ = 0 otherwise. More precisely, the transition probability between *S* and *S*′ is given by

$$\begin{array}{c} P_{S,S^{\prime }}=\{\begin{array}{cc} \frac{r\sum_{i\in S}\;w_{ij}}{w_{S}} & \text{if}\;S^{\prime }\setminus S=\left\{j\right\}\text{,}\\ \frac{\sum_{i\in V\setminus S}\;w_{ij}}{w_{S}} & \text{if}\;S\setminus S^{\prime }=\left\{j\right\}\text{,}\\ \frac{r\sum_{i,j\in S}\;w_{ij}+\sum_{i,j\in V\setminus S}\;w_{ij}}{w_{S}} & \text{if}\;S=S^{\prime }\text{,}\\ 0 & \text{otherwise,} \end{array} \end{array}$$ (4)

where *r* > 0 is the fitness and

$$\begin{array}{c} w_{S}=r\sum_{i\in S}\sum_{j\in V}\;w_{ij}+\sum_{i\in V\setminus S}\sum_{j\in V}\;w_{ij}=r\left|S\right|+N-\left|S\right| \end{array}$$ (5)

is the total reproductive weight of invaders and residents. The *fixation probability* of each subset *S* ⊂ *V* inhabited by invaders $\Phi_{S}(r)=\mathbb{P}[\;\exists n\geq 0\;:\;X_{n}=V|X_{0}=S\;]$ gives a solution of the system of 2^*N*^ linear equations

$$\begin{array}{c} \Phi_{S}\left(r\right)=\sum_{S^{\prime }}\;P_{S,S^{\prime }}\Phi_{S^{\prime }}\left(r\right). \end{array}$$ (6)

Since *G* is undirected, the only recurrent states are *S* = ∅ and *S* = *V*. Then Eq 6 has a unique solution [24]. The *(average) fixation probability* is given by

$$\begin{array}{c} \Phi \left(r\right)=\frac{1}{N}\;\sum_{i=1}^{N}\;\Phi_{\left\{i\right\}}\left(r\right). \end{array}$$ (7)

It is a rational function depending on the fitness *r* ∈ (0, +∞). Notice that Φ(*r*) may be calculated using the embedded Markov chain instead of the standard Markov chain above described, both associated to the process, making the total reproductive weight disappear from the computations [9].

### Computation method

As we proved in S1 Text, the average fixation probability is a rational function Φ(*r*) = Φ′(*r*)/Φ′′(*r*) where the numerator Φ′(*r*) = ∑_*i*_ *a*_*i*_*r*^*i*^ and the denominator Φ′′(*r*) = ∑_*i*_ *b*_*i*_*r*^*i*^ are polynomials with rational coefficients of degree less than or equal to 2^*N*^ − 2. Using the symmetries of each *ℓ*-graph, we can reduce the space of states P(V) to the set of 4-uplas

$$\left(e,k,k^{\prime },e^{\prime }\right)\in \left\{0,1\right\}\times \left\{0,1,\ldots ,n\right\}\times \left\{0,1,\ldots ,n\right\}\times \left\{0,1\right\}$$

ordered lexicographically (from halves to extra vertices) by *k* ≥ *k*′ or *e* ≥ *e*′, or equivalently the system of linear equations Eq 6 to a new system with at most

$$\begin{array}{c} \frac{{\left(2\left(n+1\right)\right)}^{2}+2\left(n+1\right)}{2}=\frac{N^{2}+N}{2}=\frac{N(N+1)}{2} \end{array}$$

linear equations. For *ℓ*_6_, *ℓ*_8_ and *ℓ*_10_, we have 21, 36 and 55 reduced states respectively. Therefore, we can lower the former bound of the degree of Φ′(*r*) and Φ′′(*r*) to a quantity

$$d=\frac{N(N+1)}{2}-2$$

proving Eq 3. Details are explained in S1 Text. Hence, we should only compute the 2(*d* + 1) coefficients involved in Φ(*r*). Actually, since Φ(*r*) converges to 1 as *r* → +∞, we can assume that *a*_*d*_ = *b*_*d*_ = 1. Thus, we can replace Eq 6 with the system of 2*d* linear equations

$$\begin{array}{c} \sum_{i=0}^{d}a_{i}r^{i}=\Phi \left(r\right)\;(\sum_{i=0}^{d}\;b_{i}r^{i}), \end{array}$$ (8)

which arise from evaluating the rational function Φ(*r*) for fitness values *r* ∈ {1, …, *d* + 1, 1/2, …, 1/*d*}. This choice is due to those are the least complex rational numbers, which can be described with only few bits, and the length in bits of the solution of Eq 8 grows exponentially depending on the coefficients [25]. Finally, we wrote a SageMath program [18] that symbolically

- computes the exact fixation probability Φ(*r*) of the graphs *ℓ*_6_, *ℓ*_8_ and *ℓ*_10_ for these fitness values, and
- solves the reduced linear system Eq 8.

This program is available at [19]. Once the fixation probability Φ of the graphs *ℓ*_6_, *ℓ*_8_ and *ℓ*_10_ has been calculated solving this system, the sign of the numerator Δ′ and the denominator Δ′′ of the rational function Δ(*r*) = Φ(*r*) − Φ_0_(*r*) is analyzed in order to prove that Δ(*r*) < 0 for all *r* > 1. The exact values of Φ and Δ are given in S1 Text.

## Supporting information

S1 Text The fixation probability as a rational function and the fixation formulas. As supporting information, we include the essential tools in order to prove that *ℓ*_6_, *ℓ*_8_ and *ℓ*_10_ are suppressors of selection for any fitness value *r* > 1. In S1 Text, we prove that the fixation probability Φ(*r*) of any connected undirected graph of order *N* is a rational function obtained as the quotient of two polynomials Φ′ and Φ′′ with rational coefficients of degree at most 2^*N*^ − 2, which is reduced to *d* for any *ℓ*-graph. Next, we give the exact values of Φ = Φ′/Φ′′ and Δ = Δ′/Δ′′ for the graphs *ℓ*_6_, *ℓ*_8_ and *ℓ*_10_.
(PDF)

S1 Fig The differences Φ_0_(*r*) − Φ(*r*) for the *ℓ*-graphs of orders between 6 and 24. (PDF)

S1 File SageMath program. To compute the fixation probabilities of *ℓ*_6_, *ℓ*_8_ and *ℓ*_10_ for any fitness value *r* > 1, we ran a SageMath program available at [19].
(SAGE)
