# Supplementary material for: Suppressors of selection
Source: PLoS One. 2017 Jul 10;12(7):e0180549. doi: 10.1371/journal.pone.0180549 (PMC5503266; doi:10.1371/journal.pone.0180549)
Supplement: S1 Text — As supporting information, we include the essential tools in order to prove that ℓ6, ℓ8 and ℓ10 are suppressors of selection for any fitness value r > 1. In S1 Text, we prove that the fixation probability Φ(r) of any connected undirected graph of order N is a rational function obtained as the quotient of two polynomials Φ′ and Φ′′ with rational coefficients of degree at most 2N − 2, which is reduced to d for any ℓ-graph. Next, we give the exact values of Φ = Φ′/Φ′′ and Δ = Δ′/Δ′′ for the graphs ℓ6, ℓ8 and ℓ10. (PDF) [file pone.0180549.s001.pdf]

Suppressors of Selection – Supporting Information – S1 Text

Fernando Alcalde Cuesta[1,2], Pablo González Sequeiros[1,3], and Álvaro Lozano Rojo[1,4,5]

[1]GeoDynApp - ECSING Group (Spain)
[2]Departamento de Matemáticas, Universidade de Santiago de Compostela, E-15782 Santiago de Compostela (Spain)
[3]Departamento de Didácticas Aplicadas, Facultade de Formación do Profesorado, Universidade de Santiago de Compostela, Avda. Ramón Ferreiro 10, E-27002 Lugo (Spain)
[4]Centro Universitario de la Defensa, Academia General Militar, Ctra. Huesca s/n. E-50090 Zaragoza (Spain)
[5]Instituto Universitario de Matemáticas y Aplicaciones, Universidad de Zaragoza, Pedro Cerbuna 12, Zaragoza (Spain)

The fixation probability is a rational function

Given a connected undirected graph $G = (V, E)$, the Markov chain associated to the invasion process is as follows: The set of states $\mathscr{S}$ of the chain is the power set of the vertex set V, where each set $S \in \mathscr{S}$ contains the nodes occupied by mutant individuals. The transition probability between two states $S, S' \in \mathscr{S}$ is

$$P_{S,S'} = \begin{cases} \frac{r\sum_{i\in S} w_{ij}}{w_S} & \text{if } S' \setminus S = \{j\}, \\ \frac{\sum_{i\in V\setminus S} w_{ij}}{w_S} & \text{if } S \setminus S' = \{j\}, \\ \frac{r\sum_{i,j\in S} w_{ij} + \sum_{i,j\in V\setminus S} w_{ij}}{w_S} & \text{if } S = S', \\ 0 & \text{otherwise,} \end{cases} \tag{1}$$

where $r > 0$ is the relative fitness for the invader mutant, d_i is the degree of the node i, and $w_{ij} = 1/d_i$ if $(i, j) \in E$ and $w_{ij} = 0$ otherwise. Notice that all the outgoing probabilities $P_{S,S'}$ have the same denominator, the *total reproductive weight of S*

$$w_S = r\sum_{i\in S}\sum_{j\in V} w_{ij} + \sum_{i\in V\setminus S}\sum_{j\in V} w_{ij} = r|S| + N - |S|.$$

Let $\Phi_S(r)$ be the probability of all population will become mutant at some point starting with mutants in the vertices belonging to S, that is, the probability of reaching the absorbing state $V \in \mathscr{S}$ starting from $S \in \mathscr{S}$. Hence, the *average fixation probability* is

$$\Phi(r) = \frac{1}{N}\sum_{i\in V} \Phi_{\{i\}}(r).$$

To compute this quantity it is enough to solve the system of linear equations

$$\Phi_S(r) = \sum_{S'\in\mathscr{S}} P_{S,S'}\Phi_{S'}(r), \tag{2}$$

with boundary conditions $\Phi_\emptyset(r) = 0$ and $\Phi_V(r) = 1$. If $\mathbf{P} = (P_{S,S'})$ is the transition matrix, Eq 2 can be written as

$$\mathbf{P}\cdot\mathbf{\Phi} = \mathbf{P}\cdot\begin{pmatrix}0\\ \mathbf{\Psi}\\ 1\end{pmatrix} = \left(\begin{array}{c|c|c} 1 & 0 & 0 \\ \hline \mathbf{b} & \mathbf{Q} & \mathbf{c} \\ \hline 0 & 0 & 1 \end{array}\right)\cdot\begin{pmatrix}0\\ \mathbf{\Psi}\\ 1\end{pmatrix} = \begin{pmatrix}0\\ \mathbf{\Psi}\\ 1\end{pmatrix} = \mathbf{\Phi}, \tag{3}$$

where $\boldsymbol{\Phi} = (0, \boldsymbol{\Psi}, 1)$ is the vector with coordinates $\Phi_S(r)$, $(1, \mathbf{b}, 0)$ is the vector with coordinates $P_{S,\emptyset}$, and $(0, \mathbf{c}, 1)$ is the vector with coordinates $P_{S,V}$. It can be rewritten as

$$(\mathbf{I} - \mathbf{Q}) \cdot \boldsymbol{\Psi} = \mathbf{c}, \tag{4}$$

where $\mathbf{I}$ is the identity matrix. It is clear that the entries of $\mathbf{I} - \mathbf{Q}$ and the coordinates of $\mathbf{c}$ belongs to $\mathbb{Q}[r]$. Therefore, Cramer's rule implies that the entries of the fundamental matrix $\mathbf{N} = (\mathbf{I} - \mathbf{Q})^{-1}$ and the coordinates of the solution $\boldsymbol{\Psi}$ of the linear system Eq 4 are rational functions on r with rational coefficients. Then,

Lemma 1. *The average fixation probability $\Phi(r)$ is a rational function on r with rational coefficients.* □

In order to bound the degree of the numerator and denominator of $\Phi(r)$, we must simplify Eq 4 as follows. If we multiply each equation by the reproductive weight w_S of the corresponding state S, we obtain the simpler equivalent linear system

$$[\mathbf{T}(\mathbf{I} - \mathbf{Q})] \cdot \boldsymbol{\Psi} = \mathbf{T} \cdot \mathbf{c},$$

where $\mathbf{T}$ is the diagonal matrix with entries w_S associated to the states $S \in \mathscr{S}$ different from $\emptyset$ and V. Obviously, all the coordinates of the matrix $\mathbf{T}(\mathbf{I} - \mathbf{Q})$ and the vector $\mathbf{T} \cdot \mathbf{c}$ are functions of the form $a\,r + b$ with a and $b \in \mathbb{Q}$. By Cramer's rule, the fixation probability of any state S is

$$\Phi_S(r) = \frac{\det [\mathbf{T}(\mathbf{I} - \mathbf{Q})]_S}{\det [\mathbf{T}(\mathbf{I} - \mathbf{Q})]}$$

where $[\mathbf{T}(\mathbf{I} - \mathbf{Q})]_S$ is the matrix obtained from $\mathbf{T}(\mathbf{I} - \mathbf{Q})$ by replacing the column associated to S with the vector $\mathbf{T} \cdot \mathbf{c}$. If the size of both matrices is $d \times d$, since each entry is a polynomial of degree at most 1, $\Phi_S(r)$ is the quotient of two polynomial with rational coefficients of degree at most d. Hence,

Lemma 2. *The average fixation probability $\Phi(r)$ is a rational function with both numerator and denominator polynomials of degree equal to the number of states minus two.*

Remark 3. A priori, the degree of the average fixation probability function is $2^N - 2$ where N is the size of the graph. However, using the symmetries of the graph it is possible to reduce the number of possible states, and hence the degree of $\Phi(r)$. For example, the average fixation probability function for the complete graph K_N has degree, at most, $N - 1$. For the complete bipartite graphs $K_{n,m}$ it would have degree $\leq (n-1)(m-1) - 2$ (see Refs. 1–3 for the concrete expressions). As we explained in the paper, for the ℓ-graphs, the degree $d = \frac{N(N+1)}{2} - 2$.

On the other hand, since the average fixation probability tends to 1 as $r \to \infty$, both numerator and denominator have the same lead coefficient, so it can be assumed to be 1.

The SageMath program

The essential tool in order to prove that ℓ_6, ℓ_8 and ℓ_{10} are suppressor of selection for any fitness value $r > 1$ is a SageMath program that symbolically

- computes the exact fixation probability $\Phi(r)$ for the graphs ℓ_6, ℓ_8 and ℓ_{10} when $r \in \{1, \dots, d+1, 1/2, \dots, 1/d\}$,
- solves the linear system

$$\sum_{i=0}^{d} a_i r^i = \Phi(r) \left(\sum_{i=0}^{d} b_i r^i \right),$$

where $\Phi'(r) = \sum_{i=0}^{d} a_i r^i$ and $\Phi''(r) = \sum_{i=0}^{d} b_i r^i$ are the numerator and the denominator of the rational function $\Phi(r)$. The symmetries of the ℓ-graphs allow to reduce the degree to $d = \frac{N(N+1)}{2} - 2 \ll 2^N - 2$. The code is available at `https://bitbucket.org/snippets/alvarolozano/7jnka/`. Next, we give the exact values of $\Phi = \Phi'/\Phi''$ and $\Delta = \Delta'/\Delta''$ for the graphs ℓ_6, ℓ_8 and ℓ_{10}.

Fixations functions for ℓ_6, ℓ_8 and ℓ_{10}

Graph ℓ_6

$$\Phi'(r) = r^{17} + \frac{20010803101}{2356885440}r^{16} + \frac{12872954057}{368903808}r^{15} + \frac{146529682470259}{1590897672000}r^{14} + \frac{20006411662050293}{114544632384000}r^{13} + \frac{57705463723529081}{229089264768000}r^{12} + \frac{9771555843017869}{34363389715200}r^{11} + \frac{1025122250624863}{4042751731200}r^{10} + \frac{60836752923313811}{343633897152000}r^{9} + \frac{9233243223215201}{98181113472000}r^{8} + \frac{1542392404026107}{42954237144000}r^{7} + \frac{3153322564729}{357951976200}r^{6} + \frac{496442293}{474304896}r^{5}$$

$$\Phi''(r) = r^{17} + \frac{22367688541}{2356885440}r^{16} + \frac{1883112090533}{42423937920}r^{15} + \frac{579196292648299}{4242393792000}r^{14} + \frac{23779172425823219}{76363088256000}r^{13} + \frac{17230349794290811}{30545235302400}r^{12} + \frac{55592085032472109}{65454075648000}r^{11} + \frac{71798191286820983}{65454075648000}r^{10} + \frac{37885437915832519}{30545235302400}r^{9} + \frac{37885437915832519}{30545235302400}r^{8} + \frac{71798191286820983}{65454075648000}r^{7} + \frac{55592085032472109}{65454075648000}r^{6} + \frac{17230349794290811}{30545235302400}r^{5} + \frac{23779172425823219}{76363088256000}r^{4} + \frac{579196292648299}{4242393792000}r^{3} + \frac{1883112090533}{42423937920}r^{2} + \frac{22367688541}{2356885440}r + 1$$

$$\Delta'(r) = r^{5}(r-1)(-\frac{1}{408}r^{13} - \frac{17959403797}{509087255040}r^{12} - \frac{2269495014623}{9163570590720}r^{11} - \frac{165933379973713}{152726176512000}r^{10} - \frac{4365681088426001}{1374535588608000}r^{9} - \frac{4487811876686051}{687267794304000}r^{8} - \frac{6774104367192077}{687267794304000}r^{7} - \frac{673669214287879}{59762416896000}r^{6} - \frac{13704670736178649}{1374535588608000}r^{5} - \frac{2361661570426121}{343633897152000}r^{4} - \frac{2506212053017441}{687267794304000}r^{3} - \frac{122207986609099}{85908474288000}r^{2} - \frac{299174081287}{818175945600}r - \frac{22137397}{474304896})$$

$$\Delta''(r) = r^{21} + \frac{22367688541}{2356885440}r^{20} + \frac{1925536028453}{42423937920}r^{19} + \frac{619458132022099}{4242393792000}r^{18} + \frac{27245137277038619}{76363088256000}r^{17} + \frac{15493177389178517}{21818025216000}r^{16} + \frac{552157240360000477}{458178529536000}r^{15} + \frac{411797892764062669}{229089264768000}r^{14} + \frac{550050599259865931}{229089264768000}r^{13} + \frac{1329324154659596831}{458178529536000}r^{12} + \frac{54074574184168127}{16969575168000}r^{11} + \frac{54074574184168127}{16969575168000}r^{10} + \frac{1329324154659596831}{458178529536000}r^{9} + \frac{550050599259865931}{229089264768000}r^{8} + \frac{411797892764062669}{229089264768000}r^{7} + \frac{552157240360000477}{458178529536000}r^{6} + \frac{15493177389178517}{21818025216000}r^{5} + \frac{27245137277038619}{76363088256000}r^{4} + \frac{619458132022099}{4242393792000}r^{3} + \frac{1925536028453}{42423937920}r^{2} + \frac{22367688541}{2356885440}r + 1$$

Graph ℓ_8

$$\Phi'(r) = r^{31} + \frac{134280899640541}{8309364148800}r^{30} + \frac{51436353602527862867}{399248328621542400}r^{29} + \frac{26139523948185254553808 7881}{3873507284286204364800 00}r^{28} + \frac{5838078795965277959077 9931453}{2236950456675283020672 0000}r^{27} + \frac{2732442855095140200328 06460287}{3441462241038896954880 0000}r^{26} + \frac{1095467949363083709179 02477449001}{5541864357182636644761 600000}r^{25} + \frac{1038203195769664571995 777599649}{2510012346740620001280 0000}r^{24} + \frac{9219730675269628384323 8979311641}{1243784941702528405340 160000}r^{23} + \frac{1174071228482583106943 0912101427861}{1018061007837995472519 16800000}r^{22} + \frac{1032937952392948232127 781620508111}{6568135534438680467865 600000}r^{21} + \frac{2080310449462551016365 104268144504161}{1099505888465035110320 70144 00000}r^{20} + \frac{7538970259827313854673 85013243433}{3739815947159983368437 760000}r^{19} + \frac{2463546677977027065113 7435076943581}{1293536339370629541553 76640000}r^{18} + \frac{1251563161394232439985 295422113577}{7853613489035965073719 296000}r^{17} + \frac{3477901185411691168774 411224802753}{2955660990497406210539 5200000}r^{16} + \frac{5510437720885200677122 578161372417}{7233591371480494146846 7200000}r^{15} + \frac{4709031129091755423187 995718697467 73}{1099505888465035110320 7014400000}r^{14} + \frac{5969238263774975752904 62451325719}{2893436548592197658738 6880000}r^{13} + \frac{7066876349108602065069 037131219649}{8457737603577193156313 08800000}r^{12} + \frac{1523065865258014338560 2731564041827}{5497529442325175551603 507200000}r^{11} + \frac{1419300274241719705184 14844712131}{1963403372258991268429 82400000}r^{10} + \frac{9117450368110736467921 34165773}{6544677907529970894766 080000}r^{9} + \frac{62491528388375488794265 01}{35414923742045297049600 0}r^{8} + \frac{11363885694605603}{10303182674104320}r^{7}$$

$$\Phi''(r) = r^{31} + \frac{142590263789341}{8309364148800} r^{30} + \frac{685751104869983351}{4697039160253440} r^{29} +$$
$$\frac{31797027234867165871976148 1}{3873507284286204364800 00} r^{28} + \frac{6140454463156862381955427241 77}{1789560365340226416537600 00} r^{27} +$$
$$\frac{7828830358350018509132270508 07}{6882924482077793909760000 0} r^{26} + \frac{2027468204090641240495620587659}{6507492237600823332864000 0} r^{25} +$$
$$\frac{623367948536553693187185508902 2867}{858988975363308679938048 00000} r^{24} + \frac{5405997383885275954244381013403073}{3681381322985608628305920 0000} r^{23} +$$
$$\frac{54122335023003970350539078924384011}{206157354087194083185131520000} r^{22} + \frac{173354579809818170060136413942072 4667}{41231470817438816637026304 00000} r^{21} +$$
$$\frac{16776173149945946521412776956946004 03}{2748764721162587775801753600 000} r^{20} + \frac{13378648977067195392365956615895582 03}{1649258832697552665481052160 000} r^{19} +$$
$$\frac{10664177881341717271399745414940451 9}{10709472939594497827799040 0000} r^{18} + \frac{93745939780908581520547461590009531 63}{8246294163487763327405260800 000} r^{17} +$$
$$\frac{3969636168792774061518691713246829 3}{3272338953764985447383040 0000} r^{16} + \frac{3969636168792774061518691713246829 3}{3272338953764985447383040 0000} r^{15} +$$
$$\frac{93745939780908581520547461590009531 63}{8246294163487763327405260800 000} r^{14} + \frac{10664177881341717271399745414940451 9}{10709472939594497827799040 0000} r^{13} +$$
$$\frac{13378648977067195392365956615895582 03}{1649258832697552665481052160 000} r^{12} + \frac{16776173149945946521412776956946004 03}{2748764721162587775801753600 000} r^{11} +$$
$$\frac{173354579809818170060136413942072 4667}{41231470817438816637026304 00000} r^{10} + \frac{54122335023003970350539078924384011}{206157354087194083185131520000} r^{9} +$$
$$\frac{5405997383885275954244381013403073}{3681381322985608628305920 0000} r^{8} + \frac{623367948536553693187185508902 2867}{858988975363308679938048 00000} r^{7} +$$
$$\frac{2027468204090641240495620587659}{6507492237600823332864000 0} r^{6} + \frac{7828830358350018509132270508 07}{6882924482077793909760000 0} r^{5} +$$
$$\frac{6140454463156862381955427241 77}{1789560365340226416537600 00} r^{4} + \frac{31797027234867165871976148 1}{3873507284286204364800 00} r^{3} +$$
$$\frac{685751104869983351}{4697039160253440} r^{2} + \frac{142590263789341}{8309364148800} r + 1$$

$$\Delta'(r) = r^{7}(r-1)(-\frac{1}{304} r^{27} - \frac{25197109151333}{398849479142400} r^{26} -$$
$$\frac{794180920248746848 69}{130663089367050240000} r^{25} - \frac{151439378562924022621961 29}{387350728428620436480000 0} r^{24} -$$
$$\frac{2366973910210561053038186471}{124943850961935807990988800} r^{23} - \frac{845531429520747906230096887311 1}{114531863381774490658406400000} r^{22} -$$
$$\frac{99152447879986011283802032820984 9}{412314708174388166370263040000 0} r^{21} - \frac{5521164844468759159410730585869037}{824629416348776332740526080000 0} r^{20} -$$
$$\frac{985364585564805190812437620862539}{610836604702797283511500800000} r^{19} - \frac{11173152912008312609935094528064451 9}{329851766539510533096210432000 00} r^{18} -$$
$$\frac{20546093708805731165320517683596132 1}{329851766539510533096210432000 00} r^{17} - \frac{11073449367030373276155500111578751 3}{109950588846503511032070144000 00} r^{16} -$$
$$\frac{15803272413831999063713425526311023 1}{109950588846503511032070144000 00} r^{15} - \frac{19980272099587550846028888561940723 7}{109950588846503511032070144000 00} r^{14} -$$
$$\frac{22453860958317641258746883635302901 1}{109950588846503511032070144000 00} r^{13} - \frac{2409466399323244638863069185204064 3}{117804202335539476105789440000 0} r^{12} -$$
$$\frac{15076786364712664621750564243779907 7}{824629416348776332740526080000 00} r^{11} - \frac{5345105200264500037417701584886285 1}{366501962821678370106900480000 0} r^{10} -$$
$$\frac{34157797385475808982809488450069283 7}{329851766539510533096210432000 00} r^{9} - \frac{6134982182129497498119956432252281}{94243361868431580884631552000 0} r^{8} -$$
$$\frac{5639056379567408901354673628901377}{1570722697807193014743859200000} r^{7} - \frac{18840773531540731523638914353570237}{109950588846503511032070144000 00} r^{6} -$$
$$\frac{2181010891816562206229565291712 33}{31414453956143860294877184000 0} r^{5} - \frac{8513611804894653231006174793127 3}{36650196282167837010690048000 0} r^{4} -$$
$$\frac{13299491130425872370185157425 1}{21575861233615288664064000 00} r^{3} - \frac{79621768891657352281009056253}{65446779075299708947660800 00} r^{2} -$$
$$\frac{56249336779522894155418 1}{35414923742045297049600 0} r - \frac{1060703020501283}{10303182674104320})$$

$$\Delta''(r) = r^{37} + \frac{142590263789341}{8309364148800} r^{36} + \frac{690448144030236791}{4697039160253440} r^{35} +$$
$$\frac{32461728426878431744799508 1}{3873507284286204364800 00} r^{34} + \frac{6403513507529948284943921381 77}{1789560365340226416537600 00} r^{33} +$$
$$\frac{10927345392515915835176820297601}{894780182670113208268800000} r^{32} + \frac{3069574771359139871534493211 97}{8837335137482599587840000} r^{31} +$$
$$\frac{348454767609907409188939226679 07}{4109995097432098947072000 00} r^{30} + \frac{4679260402378757455250126788552 6031}{2576966926089926039814144000 00} r^{29} +$$
$$\frac{5114092033360339711126949652505697 3}{14725552529194243451322368000 00} r^{28} + \frac{8272084991958763491341875459069937 21}{1374382360581293887900876800 000} r^{27} +$$
$$\frac{7889974227256354217659306239108441937}{8246294163487763327405260800 000} r^{26} + \frac{11624280269542628969132052974659677181}{8246294163487763327405260800 000} r^{25} +$$
$$\frac{1905666136325256739867011702632083 91}{98170168612949563421491200 000} r^{24} + \frac{785680055939815191787545230787594 01}{31235962740483951997747200 000} r^{23} +$$
$$\frac{10588602274956189876854293054560549 77}{34359559014532347197521920 0000} r^{22} + \frac{7383623302044652471116888965793050837}{2061573540871940831851315200 00} r^{21} +$$
$$\frac{17169655808981871074991308596300480 09}{43401548228882964881080320 0000} r^{20} + \frac{3427881858061536878224263155383490 1977}{8246294163487763327405260800 000} r^{19} +$$
$$\frac{3427881858061536878224263155383490 1977}{8246294163487763327405260800 000} r^{18} + \frac{17169655808981871074991308596300480 09}{43401548228882964881080320 0000} r^{17} +$$
$$\frac{7383623302044652471116888965793050837}{2061573540871940831851315200 00} r^{16} + \frac{10588602274956189876854293054560549 77}{34359559014532347197521920 0000} r^{15} +$$
$$\frac{785680055939815191787545230787594 01}{31235962740483951997747200 000} r^{14} + \frac{1905666136325256739867011702632083 91}{98170168612949563421491200 000} r^{13} +$$
$$\frac{11624280269542628969132052974659677181}{8246294163487763327405260800 000} r^{12} + \frac{7889974227256354217659306239108441937}{8246294163487763327405260800 000} r^{11} +$$
$$\frac{8272084991958763491341875459069937 21}{1374382360581293887900876800 000} r^{10} + \frac{5114092033360339711126949652505697 3}{14725552529194243451322368000 00} r^{9} +$$
$$\frac{4679260402378757455250126788552 6031}{2576966926089926039814144000 00} r^{8} + \frac{348454767609907409188939226679 07}{4109995097432098947072000 00} r^{7} +$$
$$\frac{3069574771359139871534493211 97}{8837335137482599587840000} r^{6} + \frac{10927345392515915835176820297601}{894780182670113208268800000} r^{5} +$$
$$\frac{6403513507529948284943921381 77}{1789560365340226416537600 00} r^{4} + \frac{32461728426878431744799508 1}{3873507284286204364800 00} r^{3} +$$
$$\frac{690448144030236791}{4697039160253440} r^{2} + \frac{142590263789341}{8309364148800} r + 1$$

Graph ℓ_{10}

$$\Phi'(r) = r^{49} +$$
$$\frac{63998337467225103992090545275949}{2453784351049966935520268248500} r^{48} +$$
$$\frac{146779191511973282687623624712046560107871 3}{434717306393948976481137730636733272 5000} r^{47} +$$
$$\frac{35863545786298851175250332766194774847587066497001}{12401832675459773375542137748470045164516250000} r^{46} +$$
$$\frac{1010722502071856154011824311272938025729799016077227537 9}{548483451904883937306726584063836217445895672500000} r^{45} +$$
$$\frac{173977361125932121188499854823535587602545984003692318940 71}{186745556243805721511575956002687093082769240875000000} r^{44} +$$
$$\frac{158482017595368473931476450856860975147350913420633382016593316 1}{407298147696748228803318189692599768070902848127968750000 0} r^{43} +$$
$$\frac{517274463035147513860432834351215563141952618120529963719956531197 7}{374714295881008370499052734517191786625230620277731250000000 0} r^{42} +$$
$$\frac{49521202955981354300030087119187885316993560807517119791866276458611 49}{116654476849271816394836680245746811467802058891725281250000000 0} r^{41} +$$
$$\frac{232819629505635204261122698697497213530179530184438758651042497936165479}{2026218618408060840738674427402803758890367851272317851562500 0000} r^{40} +$$
$$\frac{140705161942293599314085668376462617712634457962573176050687488215725213}{5078812100012722646071499687092699283413941116170792968750000000} r^{39} +$$
$$\frac{704776109036054971829235346720766616789810509486535754147964424687649474571}{117320559510293893124251642771841353446862039783545317578125000000000} r^{38} +$$
$$\frac{5609238978798902077482128976734612093504273632320828814955784214713994109652 13}{47514826601669026715321915322595748145979126112335853619140625000000000} r^{37} +$$
$$\frac{5026674984510614392865367712496407530004017342116545842037346221098565182809 09}{23757413300834513357660957661297874072989563056167926809570312500000000} r^{36} +$$
$$\frac{26429824015453356887491713705726511276974368826177265003232531171066121452 03}{760237225626704427445150645161531970335666017797373657906250000000 00} r^{35} +$$
$$\frac{20823387297836818281526008435918371919883630252711427765368887472917082446989053}{3959568883472418892943492943549645678831593842694654468261718750000000 00} r^{34} +$$
$$\frac{2182494764721817485934981785333656026352573696800474121135936965629778268351403 53}{29696766626043141697076197076622342591236953820209908511962890625000000 00} r^{33} +$$
$$\frac{12073616682732932701560441487145827994410542880111327873960403995706625725384629}{126909259085654451696906825113770694834345956496623540649414062500000000 0} r^{32} +$$
$$\frac{135789940566315840585532565857821740547661945056372353589849669605940440768457227}{1187870665041725667883047883064893703649478152808396340478515625000000000} r^{31} +$$
$$\frac{1263938665010845091528254131320795020467290201523310371883484596474040356624342127}{9898922208681047232358732358874114197078984606736636170654296875000000 00} r^{30} +$$
$$\frac{38937683392585997984347289370749507251595629992719180864625208590442969628877 53}{293446310534023139299171907871762278569535116800493167114257812500000 0} r^{29} +$$
$$\frac{12168244863533612374634189985671509035799081740724833221712831589105585405872 07}{948172625352590731068844095677597145314078985319601165771484375000000} r^{28} +$$
$$\frac{342950658867614575564774744874331500786589425104481846989084698332117314061325299}{29696766626043141697076197076622342591236953820209908511962890625000000 00} r^{27} +$$
$$\frac{136620889917538222852217823124817295526976539096842805176370201484115198050916799}{1414131744097292461765533194124873456725569229533805167236328125000000000} r^{26} +$$
$$\frac{106103728708961707725096565755154094753809519630524214452593627351709782825503313}{1414131744097292461765533194124873456725569229533805167236328125000000000} r^{25} +$$
$$\frac{496044793580204554525678229969923834502352531064764912680884504837714700470547}{918835601053315027756070454103414065322925551367880832672119140625000} r^{24} +$$
$$\frac{772359485654188724829120426699784984692635338460009360833608747322067262809039}{21519396105828363548605939910595900428432575232036165588378906250000000} r^{23} +$$
$$\frac{652516365227989567609612927273551680629147392704119960328347885247676692854839209}{29696766626043141697076197076622342591236953820209908511962890625000000 00} r^{22} +$$
$$\frac{366302474000671446200946961697442757335305613890313285591333774174703031631539261}{29696766626043141697076197076622342591236953820209908511962890625000000 00} r^{21} +$$
$$\frac{1645494418668165225995421450087912710711608999941997419463709015853368829896609}{2604979528600275587462824304966872157126048580720167413330078125000000 0} r^{20} +$$
$$\frac{34833556722763089659481832034690135656178814634338446724446742988135953855162 79}{1187870665041725667883047883064893703649478152808396340478515625000000 0} r^{19} +$$
$$\frac{363660717759324145055492057512794976245734182721607190240028880280258529672555 21}{2969676662604314169707619707662234259123695382020990851196289062500000 0} r^{18} +$$
$$\frac{1566098453886886032512195177922507302933744244599563429255680654923560486349}{3437125766903141400124559846831289651763536321783554225921630859375 00} r^{17} +$$
$$\frac{9849998529036909725835193690470491230633425936733439335488624857067513903961}{65992814724540314882391549059160761313859897378244241137695312500000 0} r^{16} +$$
$$\frac{7612244671643394966002495975771535194184958698344817283774206365930539589}{179572284964735550700385167507920438949278632321753037109375000000000} r^{15} +$$
$$\frac{1780331892413176694593527982155770314406131540035651123394684892300797027}{174046983888897533755757931584599810058531597481083712890625000000000} r^{14} +$$
$$\frac{666823956195516846359952872220117109274829807880728490758393042227}{32694089206142112098386011380595437223355235743605468750000000 0} r^{13} +$$
$$\frac{50241672766902471992514270225360386829136967407023316489284256 1}{155686139076867200468504816098073510587405884493359375000000} r^{12} +$$
$$\frac{203090003571681891310161796937656604995672476589798791 17}{534469444908586947308721574295373048214348828125000 00} r^{11} +$$
$$\frac{22310940281865417950066567363779288792016190601063 79}{753583582710229284972178509021617327704980468750 00} r^{10} +$$
$$\frac{21609304615059677300836598774088547 87}{18869431352372327272248476992968750 00} r^{9}$$

$$\Phi''(r) = r^{49} + \frac{66452121818275070927610813524449}{2453784351049966935520268248500} r^{48} +$$
$$\frac{3171065904103137883917151919165713711668601}{8694346127878979529622754612734665450000} r^{47} +$$
$$\frac{161551466808961078632134160985357629261818985772279}{49607330701839093502168550993880180658065000000} r^{46} +$$
$$\frac{2068529647022090441678077299141302609199588004130495071}{95388426418240684748995927663275863903634030000000} r^{45} +$$
$$\frac{450435638313535005425039251624357069084344950220101681650037}{3921656681119920151743095076056428954738154058375000000} r^{44} +$$
$$\frac{6867963997466308046319209185518123663135600673726493180976212363}{13625974395673031654511008527897883150008386191917500000000} r^{43} +$$
$$\frac{269061418914533471263783319921967679903930817801083532938975715087}{142748303192765093523448660768454013952468807724850000000000} r^{42} +$$
$$\frac{10872394332592777284288878621556700840415990768352322461450204898552609}{17731480481089316092015175397353515343105912951542242750000000000} r^{41} +$$
$$\frac{38291101140138887359782676914615116290259958340221391656013420168256811577}{217210635893344122127185898617580562953047433656392473687500000000000} r^{40} +$$
$$\frac{98514958000354677455643814676264534931115549410475725682221654907248284623}{217210635893344122127185898617580562953047433656392473687500000000000} r^{39} +$$
$$\frac{26732729318515826831791274995616579065637953912223549102534868243984792082723}{25341240854223480914838354838717732344522200593245788596875000000000000} r^{38} +$$
$$\frac{425183741527331139825902027524200329764363303966235727482073723400678572174677}{1900593064066761068612876612903829925839165044493434144765625000000000000} r^{37} +$$
$$\frac{828072648510561263189682099297410898323565661136075420452698337442115035231749}{1900593064066761068612876612903829925839165044493434144765625000000000000} r^{36} +$$
$$\frac{14904297300954246434065316373615468433612748954602965427264845089050815525174899}{19005930640667610686128766129038299258391650444934341447656250000000000000} r^{35} +$$
$$\frac{41550484881909446388327767743277061617985477251726998056709651669094404010048507}{316765510677793511435479435483971654306527507415572357460937500000000000} r^{34} +$$
$$\frac{22130456707641639004248146630609315027823814070629995136603247025845612921651603}{107988242276520515262095262096808518513588922982581485498046875000000000} r^{33} +$$
$$\frac{1784422236438075188537138900658095059296529954702208506947548731323074538577597981}{5939353325208628339415239415324468518247390764041981702392578125000000000} r^{32} +$$
$$\frac{3287151019212848217807984745591777366372276860411583588494571887934733431113199393}{7919137766944837785886985887099291357663187685389308936523437500000000000} r^{31} +$$
$$\frac{12891830160861887042312930556116527397598369437831253112410533891470144462896563509}{2375741330083451335766095766129787407298956305616792680957031250000000000} r^{30} +$$
$$\frac{1143364706849144471368732701620706675895368660811433194915842968849736588740046217}{169695809291675095411863983294984814807068307544056620068359375000000000} r^{29} +$$
$$\frac{1894223361894024164709180344193508276149281595591591986658211519315537547323846207}{2375741330083451335766095766129787407298956305616792680957031250000000000} r^{28} +$$
$$\frac{21428097029880373218311112435358229670656891082066760972501955864501432138378896531}{2375741330083451335766095766129787407298956305616792680957031250000000000} r^{27} +$$
$$\frac{801022019708817904455746198578617530054038040854412364759411591253792343583646439}{81922114830463839164348129866544393355136424331613540722656250000000000} r^{26} +$$
$$\frac{6043831092625088541215047733673129681002510108096187925439945057336154334932897427}{5939353325208628339415239415324468518247390764041981702392578125000000000} r^{25} +$$
$$\frac{6043831092625088541215047733673129681002510108096187925439945057336154334932897427}{5939353325208628339415239415324468518247390764041981702392578125000000000} r^{24} +$$
$$\frac{801022019708817904455746198578617530054038040854412364759411591253792343583646439}{81922114830463839164348129866544393355136424331613540722656250000000000} r^{23} +$$
$$\frac{21428097029880373218311112435358229670656891082066760972501955864501432138378896531}{2375741330083451335766095766129787407298956305616792680957031250000000000} r^{22} +$$
$$\frac{1894223361894024164709180344193508276149281595591591986658211519315537547323846207}{2375741330083451335766095766129787407298956305616792680957031250000000000} r^{21} +$$
$$\frac{1143364706849144471368732701620706675895368660811433194915842968849736588740046217}{169695809291675095411863983294984814807068307544056620068359375000000000} r^{20} +$$
$$\frac{12891830160861887042312930556116527397598369437831253112410533891470144462896563509}{2375741330083451335766095766129787407298956305616792680957031250000000000} r^{19} +$$
$$\frac{3287151019212848217807984745591777366372276860411583588494571887934733431113199393}{7919137766944837785886985887099291357663187685389308936523437500000000000} r^{18} +$$
$$\frac{1784422236438075188537138900658095059296529954702208506947548731323074538577597981}{5939353325208628339415239415324468518247390764041981702392578125000000000} r^{17} +$$
$$\frac{22130456707641639004248146630609315027823814070629995136603247025845612921651603}{107988242276520515262095262096808518513588922982581485498046875000000000} r^{16} +$$
$$\frac{41550484881909446388327767743277061617985477251726998056709651669094404010048507}{316765510677793511435479435483971654306527507415572357460937500000000000} r^{15} +$$
$$\frac{14904297300954246434065316373615468433612748954602965427264845089050815525174899}{19005930640667610686128766129038299258391650444934341447656250000000000000} r^{14} +$$
$$\frac{828072648510561263189682099297410898323565661136075420452698337442115035231749}{1900593064066761068612876612903829925839165044493434144765625000000000000} r^{13} +$$
$$\frac{425183741527331139825902027524200329764363303966235727482073723400678572174677}{1900593064066761068612876612903829925839165044493434144765625000000000000} r^{12} +$$
$$\frac{26732729318515826831791274995616579065637953912223549102534868243984792082723}{25341240854223480914838354838717732344522200593245788596875000000000000} r^{11} +$$
$$\frac{98514958000354677455643814676264534931115549410475725682221654907248284623}{217210635893344122127185898617580562953047433656392473687500000000000} r^{10} +$$
$$\frac{38291101140138887359782676914615116290259958340221391656013420168256811577}{217210635893344122127185898617580562953047433656392473687500000000000} r^{9} +$$
$$\frac{10872394332592777284288878621556700840415990768352322461450204898552609}{17731480481089316092015175397353515343105912951542242750000000000} r^{8} +$$
$$\frac{269061418914533471263783319921967679903930817801083532938975715087}{142748303192765093523448660768454013952468807724850000000000} r^{7} +$$
$$\frac{6867963997466308046319209185518123663135600673726493180976212363}{13625974395673031654511008527897883150008386191917500000000} r^{6} +$$
$$\frac{450435638313535005425039251624357069084344950220101681650037}{3921656681119920151743095076056428954738154058375000000} r^{5} +$$
$$\frac{2068529647022090441678077299141302609199588004130495071}{95388426418240684748995927663275863903634030000000} r^{4} +$$
$$\frac{161551466808961078632134160985357629261818985772279}{49607330701839093502168550993880180658065000000} r^{3} +$$
$$\frac{3171065904103137883917151919165713711668601}{8694346127878979529622754612734665450000} r^{2} +$$
$$\frac{66452121818275070927610813524449}{2453784351049966935520268248500} r + 1$$

$$
\begin{aligned}
\Delta'(r) = (r-1)r^9\Big(&-\tfrac{81}{26800}r^{45} - \\
&\frac{563319538758565590920518332973}{6524782699847965260642688600000}r^{44} - \\
&\frac{185118512906802958216749583829138613138971}{150272649123834214092245141454673230000000}r^{43} - \\
&\frac{11292851072467750513499229152350823090599713979537}{964586985869093484764388491547670179462375000000}r^{42} - \\
&\frac{27951381645948785379888787463235668294504668152854207}{335217853505001795200297386666566567318112500000000}r^{41} - \\
&\frac{60827942349203178778068863211998358870186907289615783102254391}{128107451583250724956941105817843345854779699240250000000000}r^{40} - \\
&\frac{8562912202100990311905987439041329717463210575569086154366093523}{3801449378502983468830969770464264501995093249194375000000000}r^{39} - \\
&\frac{44621655705123136077347662614843260751907658760768760237116789155653}{4871285846453108816487685548723493226127998063610506250000000000}r^{38} - \\
&\frac{80492686432889156295605195823804626469146709501499514533353144038439997}{24683026806061832059907488479270518517391753824590053828125000000000}r^{37} - \\
&\frac{2157246710904829367097195187318479384875565969609496310367800179931386703}{20885638066667704050690951790151977207023791697730045546875000000000}r^{36} - \\
&\frac{23941595327872587256493987343365062085807713765396772351115918617324986847}{81221925814818849086020368072813244693981412157839066015625000000000}r^{35} - \\
&\frac{28641097615802981958553267469679468109543713475889363519184564264830177497}{3741324929265277694119835852172893554801505993097311308593750000000000}r^{34} - \\
&\frac{1823892724320361051151326792463966345135150426030137569700252876097945396887}{10003121389825058255857245331072789083364026549965442867187500000000000}r^{33} - \\
&\frac{70499700652415621486993348713301146159860420872071990636425357270453364200547}{175980839265440839686377464157762030170293059675317976367187500000000000}r^{32} - \\
&\frac{1937320728607288174042845117274152056881858558885139395629838587246738597971278 1}{2375741330083451335766095766129787407298956305616792680957031250000000000}r^{31} - \\
&\frac{183229707773937520910155652073606115259697013470625694328906663299547816303896081}{1187870665041725667883047883064893703649478152808396340478515625000000000000}r^{30} - \\
&\frac{80690365377769302841410373659439059544672078289902985512877385073728956242240459}{29696766626043141697076197076622342591236953820209908511962890625000000000}r^{29} - \\
&\frac{265118588319449904088785046907749369413286006479697580445186959644220145865382569}{59393533252086283394152394153244685182473907640419817023925781250000000000}r^{28} - \\
&\frac{208357604885427163154003094265059665124071798476776960396417115314565417433}{304331109623322516929120505968381398028364839680590820312500000000000}r^{27} - \\
&\frac{19426468826311685890111431991671272181216667262683895205966558507624928619658101}{19797844417362094464717464717748228394157969213473272341308593750000000000}r^{26} - \\
&\frac{6174768978107861419038912615027599943737329385627295727458353708683921405584637}{46951409685443702287867505259481964571125618688078906738281250000000000}r^{25} - \\
&\frac{1959429974773129382885912492383125905939334993697276431408852173926975516152184711}{1187870665041725667883047883064893703649478152808396340478515625000000000000}r^{24} - \\
&\frac{20012471149919090534576487714214848267424443570957198256689224909784074027410747}{1032931013079761450333085115708603220564763611137735948242187500000000000}r^{23} - \\
&\frac{110121595804291167778052089636529865845490314087034606537101778851663521743695293}{5164655065398807251665425578543016102823818055688679741210937500000000000}r^{22} - \\
&\frac{326615826789904548993876020347565973369126840229159564065219595833069862596943361}{14848383331302157084853809853831117129561847691010495425598144531250000000000}r^{21} - \\
&\frac{289509871858714149262817301609610759031626458944642575540032303015534612546931}{1360676592258563193451372145549706418842472110891633837890625000000000}r^{20} - \\
&\frac{17630909215964265182870222431299317526595637721076822566564048965898951483251201}{913746665416712052217729140819149002807290886775689492675781250000000000}r^{19} - \\
&\frac{54105352084418197792331252631216415905145343333898279336690202899776497173666487}{3299640736227015744119577452958038065692994868912212056884765625000000000}r^{18} - \\
&\frac{1549649686748338374454987051806469178896730084238877170009170310032316093437520631}{1187870665041725667883047883064893703649478152808396340478515625000000000000}r^{17} - \\
&\frac{1135420222193602957320608891428099418884365703673049006806329037681831956344373}{117031592614948341663354471237920561935909177616590772460937500000000000}r^{16} - \\
&\frac{11330668168315445571882115643884537226967185502728708803240987316670851550989}{1683704929826261382380190051260639400787342706422866210937500000000000}r^{15} - \\
&\frac{84578352318555854318068425149237494160595130472327482696493859485784343700267}{1948287133084673885325648487887311306625353703146459472656250000000000}r^{14} - \\
&\frac{5136997041921364612112546442178345709278066935905959887612494862149982516339931}{1979784441736209446471746471774822839415796921347327234130859375000000000}r^{13} - \\
&\frac{2178334152914974787743317152025992846465590849994367955140829072881017947763137}{152291110902785342036288190136524833801215147795948248779296875000000000}r^{12} - \\
&\frac{39661125052776054791815313579720169806017945409553794150895806492299908416797}{5484167428632159131500682747298678225528523327831931396484375000000000}r^{11} - \\
&\frac{1099102683812100152919952408177060036078549127470602338328818688702513211800499}{3299640736227015744119577452958038065692994868912212056884765625000000000}r^{10} - \\
&\frac{5489104698106140057625570314166626863533545889798027408520383285191057226547333}{3959568883472418892943492943549645678831593842694654468261718750000000000}r^{9} - \\
&\frac{613110683251160291925645271049607211243871882884379663696704067168400911092199}{1187870665041725667883047883064893703649478152808396340478515625000000000}r^{8} - \\
&\frac{26894143058737575665255926430243645704851982037548246998334833392541589960819}{158382755338896755717739717741985827153263753707786178730468750000000000}r^{7} - \\
&\frac{164922623283682397754680273310812198254876823713073900932151067207404659961}{339391618583350190823727966589969629614136615088113240136718750000000000}r^{6} - \\
&\frac{206252898674636190464249334702901618990003672145157753323380738467272477}{17404698388889753375575793158459981005853159748108371289062500000000}r^{5} - \\
&\frac{352291059682303397502433427535140117512763041819685007703823551579}{14712340142763950444273705121267946750509856084622460937500000000}r^{4} - \\
&\frac{29962903292647976025325503049075929992044225870272449515704223}{778430695384336002342524080490367552937029422466796875000000}r^{3} - \\
&\frac{239277682879825336161377672215173406914951652136521829783}{5184353615613293388894599270665118567679183632812500000}r^{2} - \\
&\frac{34572296001809112450941603979281195831409275758813}{9419794783877866062152231362770216596312255859375}r - \\
&\frac{273987326268735002858812178111979787}{188694313523723272722484769929687500 0}\Big)
\end{aligned}
$$

$$\Delta''(r) = r^{57} +$$
$$\frac{66452121818275070927610813524449}{2453784351049966935520268248500} r^{56} +$$
$$\frac{3179760250231016863446774673778448377118601}{869434612787897952962275461273466545000 0} r^{55} +$$
$$\frac{1628949069452010463740469486617692255914625199822 79}{49607330701839093502168550993880180658065000000} r^{54} +$$
$$\frac{483785634994582790704969970467916746580214541569221948 33}{2193933807619535749226906336255344869783582690000000} r^{53} +$$
$$\frac{2316565641074041276575334998763454284500108942441088830795 31}{196082834055996007587154753802821447736907702918750000 0} r^{52} +$$
$$\frac{716843116072056855305629840992648505651790742013159770253526 7113}{136259743956730316545110085278978831500083861919175000000 00} r^{51} +$$
$$\frac{2859260167815524917572517587129656270950916109020546596271655 71887}{142748303192765093523448660768454013952468807724850000000 0000} r^{50} +$$
$$\frac{118052222795373448700980947336404343408206660609340472704811192 07967299}{177314804810893160920151753973535153431059129515422427500 0000000} r^{49} +$$
$$\frac{294081709734604509298806812052828737227848392311211964140331004 6791028651}{149800438547133877329093723184538319277963747349236188750 0000000000} r^{48} +$$
$$\frac{188293921867802609980121265755191801371358726341075995041060481 77001370983}{362017726488906870211976497695967604921745722760654122812 500000000000} r^{47} +$$
$$\frac{475614070681131714206964185084515330531750463689113956780689683 00303173325997}{380118612813352213722575322580765985167833008898686828953 125000000000} r^{46} +$$
$$\frac{524037357309638139993594284647384667702228606494496816682135156 81834670733 5427}{190059306406676106861287661290382992583916504449343414476562 5000000000} r^{45} +$$
$$\frac{845931344738732607494697405805997243779658709135902144065789551 2613663035609}{150840719370377862588323540706653168717394051150272551171875 00000000} r^{44} +$$
$$\frac{287775124630634889566487180871029887926987775953323161632727137 0672552413629167}{271513294866680152658982373271975703691309292070490592109375 000000000000} r^{43} +$$
$$\frac{257875673043496133768272776258615935311252015990350644772705147 5200597178416409}{137724135077301526711078015427813762741968481485031459765625 0000000000000} r^{42} +$$
$$\frac{492369024007770206158782745957266597900056372641367109988420049 606172193809987419}{158382755338896755717739717741985827153263753707786178730468 750000000000} r^{41} +$$
$$\frac{827254739411915971204146165247084942401593952605011862683522913 5981007544672562 91}{169695809291675095411863983294984814807068307544056620068359375 000000000000} r^{40} +$$
$$\frac{861621055408051894593309949945936485336301252911211142913102886 7463619051637282857}{118787066504172566788304788306489370364947815280839634047851562 5000000000} r^{39} +$$
$$\frac{244315156207566837311212145667003563077782129448668020428560900 0353452413777580783 3}{237574133008345133576609576612978740729895630561679268095703125 0000000000} r^{38} +$$
$$\frac{552196271145602876042689106655107647236337977956942996035315489 39614579223881344 17}{395956888347241889294349294354964567883159384269465446826171875 000000000} r^{37} +$$
$$\frac{215615649511679197508324874027757668202653365414278130683009704 0345127109261937088 9}{118787066504172566788304788306489370364947815280839634047851562 5000000000} r^{36} +$$
$$\frac{180094645405691272853633604334198126975439052098418486917560976 913397771524964522 61}{791913776694483778588698588709929135766318768538930893652343750 00000000} r^{35} +$$
$$\frac{653176776632533571518945119402096783891933191912134904390590040 314598763551248022 59}{237574133008345133576609576612978740729895630561679268095703125 0000000000} r^{34} +$$
$$\frac{636172340246570460139100897402083527187001353688207725821833063 89360497380478403 43}{197978444173620944647174647177482283941579692134732723413085937 500000000} r^{33} +$$
$$\frac{332218137183117318606269693439063144199440187806610451102175232 25239389766962597 67}{913746665416712052217729140819149002807290886775689492675781250 00000000} r^{32} +$$
$$\frac{411746169241143538543365892123025575338634897964453420637348649 86046321729450642 69}{103293101307976145033308511570860322056476361113773594824218750 000000000} r^{31} +$$
$$\frac{100667123751738575301792677126882266925325220092976644231277321 3248315473781712585 49}{237574133008345133576609576612978740729895630561679268095703125 0000000000} r^{30} +$$
$$\frac{582392814179375481810561191882467076264096587578594028325974124 56373577529537229}{133318817625333969459376866786183356189615954299483315429687500 0000000} r^{29} +$$
$$\frac{582392814179375481810561191882467076264096587578594028325974124 56373577529537229}{133318817625333969459376866786183356189615954299483315429687500 0000000} r^{28} +$$
$$\frac{100667123751738575301792677126882266925325220092976644231277321 3248315473781712585 49}{237574133008345133576609576612978740729895630561679268095703125 0000000000} r^{27} +$$
$$\frac{411746169241143538543365892123025575338634897964453420637348649 86046321729450642 69}{103293101307976145033308511570860322056476361113773594824218750 000000000} r^{26} +$$
$$\frac{332218137183117318606269693439063144199440187806610451102175232 25239389766962597 67}{913746665416712052217729140819149002807290886775689492675781250 00000000} r^{25} +$$
$$\frac{636172340246570460139100897402083527187001353688207725821833063 89360497380478403 43}{197978444173620944647174647177482283941579692134732723413085937 500000000} r^{24} +$$
$$\frac{653176776632533571518945119402096783891933191912134904390590040 314598763551248022 59}{237574133008345133576609576612978740729895630561679268095703125 0000000000} r^{23} +$$
$$\frac{180094645405691272853633604334198126975439052098418486917560976 913397771524964522 61}{791913776694483778588698588709929135766318768538930893652343750 00000000} r^{22} +$$
$$\frac{215615649511679197508324874027757668202653365414278130683009704 0345127109261937088 9}{118787066504172566788304788306489370364947815280839634047851562 5000000000} r^{21} +$$
$$\frac{552196271145602876042689106655107647236337977956942996035315489 39614579223881344 17}{395956888347241889294349294354964567883159384269465446826171875 000000000} r^{20} +$$
$$\frac{244315156207566837311212145667003563077782129448668020428560900 0353452413777580783 3}{237574133008345133576609576612978740729895630561679268095703125 0000000000} r^{19} +$$
$$\frac{861621055408051894593309949945936485336301252911211142913102886 7463619051637282857}{118787066504172566788304788306489370364947815280839634047851562 5000000000} r^{18} +$$
$$\frac{827254739411915971204146165247084942401593952605011862683522913 5981007544672562 91}{169695809291675095411863983294984814807068307544056620068359375 000000000000} r^{17} +$$
$$\frac{492369024007770206158782745957266597900056372641367109988420049 606172193809987419}{158382755338896755717739717741985827153263753707786178730468 750000000000} r^{16} +$$
$$\frac{257875673043496133768272776258615935311252015990350644772705147 5200597178416409}{137724135077301526711078015427813762741968481485031459765625 0000000000000} r^{15} +$$
$$\frac{287775124630634889566487180871029887926987775953323161632727137 0672552413629167}{271513294866680152658982373271975703691309292070490592109375 000000000000} r^{14} +$$
$$\frac{845931344738732607494697405805997243779658709135902144065789551 2613663035609}{150840719370377862588323540706653168717394051150272551171875 00000000} r^{13} +$$
$$\frac{524037357309638139993594284647384667702228606494496816682135156 81834670733 5427}{190059306406676106861287661290382992583916504449343414476562 5000000000} r^{12} +$$
$$\frac{475614070681131714206964185084515330531750463689113956780689683 00303173325997}{380118612813352213722575322580765985167833008898686828953 125000000000} r^{11} +$$
$$\frac{188293921867802609980121265755191801371358726341075995041060481 77001370983}{362017726488906870211976497695967604921745722760654122812 500000000000} r^{10} +$$

$$\frac{294081709734604509298806812052828737227848392311211964140331004679102865 1}{149800438547133877329093723184538319277963747349236188750000000000} r^9 + \frac{1180522227953734487009809473364043434082066606093404727048111920796729 9}{177314804810893160920151753973535153431059129515422427500000000 0} r^8 + \frac{2859260167815524917572517587129656270950916109020546596271655718 87}{142748303192765093523448660768454013952468807724850000000000} r^7 + \frac{716843116072056855305629840992648505651790742013159770253526711 3}{136259743956730316545110085278978831500083861919175000000000} r^6 + \frac{231656564107404127657533499876345428450010894244108883079531}{196082834055996007587154753802821447736907702918750000 00} r^5 + \frac{483785634994582790704969970467916746580214541569221948 33}{21939338076195357492269063362553448697835826900000 00} r^4 + \frac{1628949069452010463740469486617692255914625199822 79}{496073307018390935021685509938801806580650000 00} r^3 + \frac{317976025023101686344677467377844837711860 1}{869434612787897952962275461273466545000 0} r^2 + \frac{6645212181827507092761081352444 9}{245378435104996693552026824850 0} r + 1$$

References

1. P. A. P. Moran, Random processes in genetics. *Proc. Cambridge Philos. Soc.* **54**, 60–71 (1958).
2. B. Voorhees and A. Murray, Fixation probabilities for simple digraphs. *Proceedings of the Royal Society of London A: Mathematical, Physical and Engineering Sciences* **469** (2013).
3. T. Monk, P. Green, and M. Paulin, Martingales and fixation probabilities of evolutionary graphs. *Proceedings of the Royal Society of London A: Mathematical, Physical and Engineering Sciences* **470** (2014).
