# Supplementary figures and images for: Suppressors of selection
Source: PLoS One. 2017 Jul 10;12(7):e0180549. doi: 10.1371/journal.pone.0180549 (PMC5503266; doi:10.1371/journal.pone.0180549)

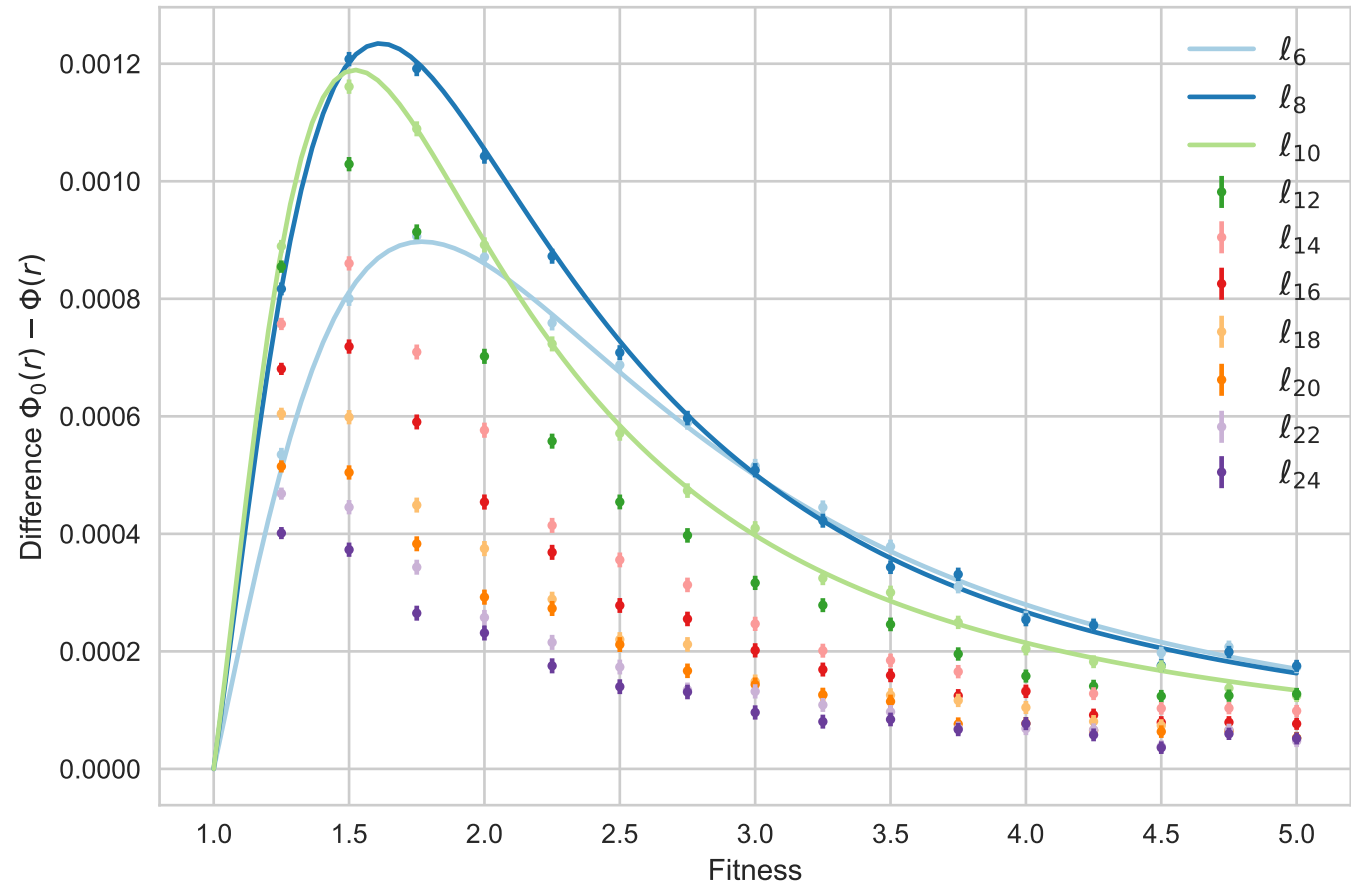

Supplement: S1 Fig — (PDF) [file pone.0180549.s002.pdf]
